# Association between type 1 diabetes mellitus and epilepsy: more than coincidence?

**DOI:** 10.1186/1758-5996-7-S1-A210

**Published:** 2015-11-11

**Authors:** Tiago Silva Aguiar, Joana Rodrigues Dantas Vezzani, Débora Batista de Araújo, Soniza Vieira Alves-Leon, Lenita Zajdenverg, Melanie Rodacki, Marco Antônio Salles Dantas de Lima

**Affiliations:** 1 Universidade Federal do Rio de Janeiro, Rio de Janeiro, Brazil

## Background

Type 1 diabetes mellitus (T1D) patients have an increased risk of seizures at extremes of glycemic control. There is growing evidence about the role of autoimmunity in the epileptogenesis and glutamic acid decarboxylase antibodies (GAD-ab), a well known antibody related to neurologic diseases, can be a link that justifies epilepsy in T1D population. GAD catalyzes the conversion of glutamic acid, the main excitatory central nervous system (CNS) amino acid, into gamma-aminobutyric acid (GABA), the main inhibitory CNS neurotransmitter. GABA-secreting neurons and pancreatic beta cells are the major cells expressing GAD. Seizures may be result of imbalance between excitation and inhibition determined by inhibition of GAD activity caused by autoantibodies. The potential pathogenic role of GAD-ab in neurological disorders is not fully understood, but inhibition of GABA synthesis or interfering with the exocytosis are plausible hypotheses. The spectrum of neurological disorders associated with GAD-ab includes cerebellar ataxia, myoclonus palatal, limbic encephalitis, encephalomyelitis, stiff person syndrome and others.

## Objectives

Assess the prevalence of epilepsy in T1D population.

## Materials and methods

Cross-sectional study in which data were collected on all T1D patients who had attended the Type 1 Diabetes Clinic. Epilepsy was diagnosed when two or more unprovoked euglycemic seizure had been reliably documented.

## Results

Data were analyzed for 375 T1D patients (165 males and 210 females, mean age 28.0±10,9 yrs., range 11-66 yrs.), of these 17 had a confirmed diagnosis of epilepsy (9 males and 8 females, mean age 27,1±6,9 range 17-38 yrs.). The frequency of epilepsy in this T1D population is 4,5%.

## Conclusion

We found an increased frequency of epilepsy in T1D. The prevalence of epilepsy in general population is between 0,5-1%, but in observed T1D population the frequency of epilepsy is 4,5%, which is 4,5-9 times higher. The reason of this association is not completely understood, but metabolic alterations and autoimmunity can play a role. In the last few yrs., there has been increasing interest in the potential role of GAD-ab in the pathogenesis of several neurologic diseases, including epilepsy. Epilepsy and T1D are serious worldwide problems with potential morbidity and social management costs. This possible association may result in different therapeutic strategies based on its possible autoimmune process.

